# Demographics and Incidence of Histologically Confirmed Intracranial Tumors: A Five-year, Two-center Prospective Study

**DOI:** 10.7759/cureus.1476

**Published:** 2017-07-16

**Authors:** George S Stoyanov, Deyan L Dzhenkov, Martina Kitanova, Peter Ghenev, Anton B Tonchev

**Affiliations:** 1 Department of Physiology and Pathophysiology, Division of Pathophysiology, Faculty of Medicine, Medical University – Varna “Prof. Dr. Paraskev Stoyanov”, Varna, Bulgaria; 2 Department of General and Clinical Pathology, Forensic Medicine and Deontology, Faculty of Medicine, Medical University – Varna “Prof. Dr. Paraskev Stoyanov”, Varna, Bulgaria; 3 Department of Anatomy and Cell Biology, Faculty of Medicine, Medical University – Varna “Prof. Dr. Paraskev Stoyanov”, Varna, Bulgaria

**Keywords:** cns, intracranial tumors, intracranial metastases, non-tumor volume occupying lesions, descriptive statistics, cancer epidemiology, cancer demographics

## Abstract

Introduction

Intracranial tumors (ICTs) are a diverse group of malignancies that pose an immediate threat to patients' lives, no matter their local or metastatic origin, benign or malignant nature. These lesions have severe clinical courses and need to be diagnosed and treated as soon as possible, with pathological verification being the pivotal moment in the process of determining curative modalities.

Aim

The aim of this study was to compare the incidence of histologically confirmed ICTs in Eastern Bulgaria, based on their type (primary, metastatic, and non-volume occupying lesions (NVOL)), their respective subtypes, and incidence in a descriptive manner.

Materials and Methods

For a period of five full calendar years (January 1st, 2012 – December 31st, 2016), all histologically confirmed cases of intracranial tumors were prospectively collected from two individual tertiary healthcare institutions. The cases were then statistically analyzed in a descriptive manner, and incidences of primary, metastatic, and NVOL were compared with regards to their specific origins, types, and subtypes. Metastatic tumors were further segregated relative to their intracranial metastatic location.

Results

The total number of individual ICTs registered in the set timeframe was 822. Primary ICTs represented a total of 66.12% of the histologically confirmed cases, with the most common entries being tumors from a glial and meningeal origin, 30.90% were histologically confirmed as metastatic ICTs, from which the most common entries were of pulmonary origin, and the other 2.94% were NVOL. On behalf of their intracranial metastatic location, metastatic tumors were located predominantly in the supratentorial region, represented as a total of 87.80%, while the other 12.20% were located in the subtentorial region. Based on the descriptive analysis, the annual incidence per 100,000 capita of all ICTs is 9.12, comprised of 6.03 per 100,000 for primary ICTs, 2.82 per 100,000 for metastatic ICTs, and 0.27 per 100,000 for NVOL. The annual incidence of the most commonly diagnosed primary ICTs per 100,000 is 2.36 for meningioma, 2.03 for glioblastoma, and 0.48 for pituitary adenoma. The annual incidence of the most commonly diagnosed metastatic ICTs per 100,000 is 1.32 for lung cancer metastases, 0.28 for gastrointestinal tract (GIT) metastases, 0.22 for melanoma, and 0.17 for breast cancer metastases.

Conclusion

Based on our results, primary ICTs are operated and biopsied more than two times as much as metastatic ICTs and only a small fraction of neurosurgical interventions are undertaken due to NVOL. Metastatic ICTs are predominantly supratentorial with no evidence of a tumor predominantly metastasizing in the subtentorial region. The demographics reported in the study establish some aspects of age and gender preferences, as well as the annual incidence per 100,000 for the most commonly diagnosed types of ICTs in our population.

## Introduction

Intracranial tumors (ICTs) are either signs of advanced distant malignant processes or local processes that can be either benign, malignant, or reactive in nature since non-tumor volume-occupying lesions (NVOL) can be viewed as distinct types of specific intracranial processes [1-2]. These lesions present an immediate threat to patients' lives and need to be diagnosed and treated as soon as possible, regardless of their origin [3-5]. Irrespective of the differences in the distinct tumor groups and the involvement of many medical specialists, these tumors are hard to diagnose and treat due to their state of advancement, biological properties, and the specifics of the blood-brain barrier [6-8].

Despite variances in the pathogenesis, evolution of ICT groups, and even within a clinical ICT entity, the primary symptom and reason for the diagnosis of an ICT is almost always the onset of neurological deficits [9-11]. The first sign of a distant malignant process can rarely be a neurological deficit caused by its intracranial spread, while, in most other cases, these metastases are the first sign of advancement of a condition and are difficult or resistant to treatment [12-13]. In the case of local tumors, the neurological deficit is virtually always the first sign of the disease, and its rate, measured via the Karnofski neurological status score, can be used as a predictive factor [14]. Primary ICTs seldom have a prior generalized manifestation, such as the case with some astrocytomas that can cause epileptic seizures and pituitary adenomas, which can cause a variety of conditions, such as gigantism, acromegaly, Cushing’s disease, or generalized panhyperpituitarism [15].

Local and metastatic ICTs without a systemic or neurological manifestation are discovered rarely as incidental findings when the patient undergoes radiological investigation in the head and neck region for non-related reasons [16-18]. While metastatic disease is often associated with an advanced age and some local tumors are generally reserved for younger individuals, both groups can be observed virtually in all age groups [13, 19-20]. This can sometimes pose as a difficulty for both the clinical and pathological diagnosis, especially in the case of severely anaplastic tumors, even with the help of a wide range of immunohistochemical markers [21].

Because of the different etiology and pathogenesis of distinct groups of tumors, few statistical studies have been targeted at comparing their incidence altogether. Studies targeted at metastatic tumors are mainly focused on the radiological and neurological or autopsy findings and completely exclude primary tumors from the reports [13, 20, 22-24]. On the other hand, studies targeted at primary ICTs exclude metastatic lesions [19, 25]. The different approaches in diagnosis and statistical analysis seemingly restrict the opportunities for the two groups to be statistically compared, allowing for a wide interpretation of their incidences, respective to the design of individual studies with only a few available studies directly comparing incidence between the two main groups but excluding NVOLs, to our knowledge [1-2, 13, 19-20, 23-28].

The aim of this study was to compare the incidence and demographics of histologically confirmed ICTs (primary, metastatic, and NVOLs) in Eastern Bulgaria with regard to their respective types and subtypes in a descriptive manner, based on the total number of individual cases.

## Materials and methods

We prospectively collected medical data for all pathologically confirmed ICT cases for the span of five calendar years (January 1st, 2012 – December 31st, 2016) from the central databases and pathological archives of two tertiary health centers - St. Marina University Hospital, Varna, Bulgaria and St. Anna Hospital, Varna, Bulgaria. The neurosurgery wards in the two respective centers service the whole northeastern and part of the southeastern region of Bulgaria, providing for the neurosurgical needs of nearly one-third of the country’s total population (1,802,793 people in total). The Committee on Ethics for Scientific Research, Medical University - Varna approved this study, protocol #20 (1).

The main criteria for what was deemed a primary tumor were set accordingly to the 2016 edition of the World Health Organization (WHO) classification of tumors of the central nervous system (CNS) [29]. Metastatic ICT histological findings, including the wide assay of immunohistochemical profiles, radiological findings, and medical documentation, were reviewed to pinpoint the primary location. For some metastatic ICTs, such data could not be retrieved, and thus their primary location remained statistically unknown.

A case was regarded as an individual patient with a histologically confirmed intracranial process, not an individual pathological finding. This avoided statistical blurring from patients operated or biopsied more than once in either of the institutions. Therefore, despite the number of histologically confirmed findings being more than a thousand, the total number of individual cases reviewed for the aim of this study was 822.

The criteria for the descriptive statistical segregation of these 822 cases were primarily the place of origin – metastatic, primary intracranial, or NVOL, the main histological type, cellular origin, and in the case of metastatic tumors, the location of origin and intracranial location (sub or supratentorial), patient age, and gender [30].

The broad term “primary ICT” was chosen in favor of “CNS tumor” with the idea of avoiding the common misclassification of tumors from non-neuroectodermal origin into this group and the goal of classifying only tumors developing inside the cranial cavity. NVOLs were viewed as lesions originating inside of the cranial cavity but reactive in nature. A metastatic lesion was deemed as a malignant formation with a primary origin other than the CNS and cranial cavity. The criteria also excluded all cases of CNS tumors with a primary localization outside of the cranial cavity, such as spinal cord lesions.

Statistical analysis was carried out with MaxStat Pro version 3.6 (http://www.maxstat.de/) using a descriptive statistical approach. All figures were generated with the built-in capabilities of Microsoft Office 2016 (Microsoft Corp., Redmond, WA).

## Results

**Figure 1:**
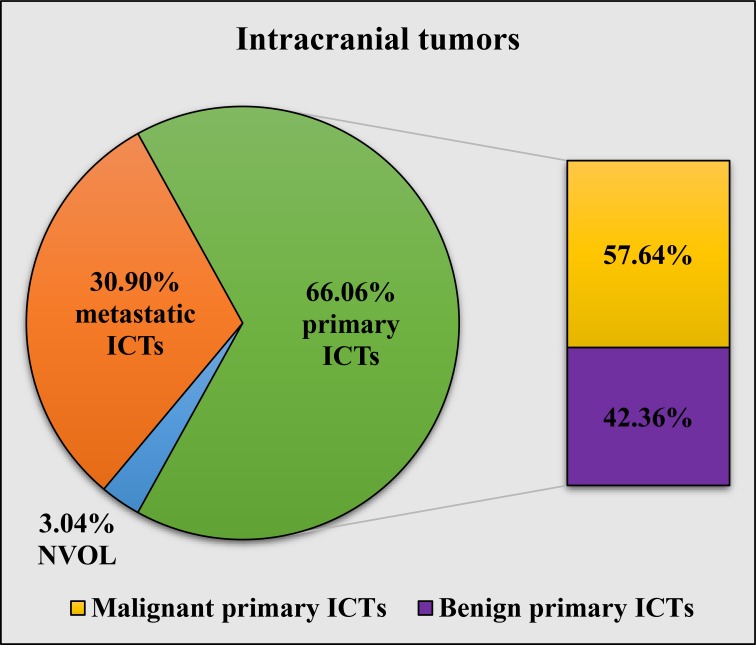
Incidence of histologically confirmed benign, malignant, and primary ICTs, as well as metastatic ICTs and NVOL ICTs: intracranial tumors; NVOL: non-volume ocupying lesions

Statistical analysis of the 822 histologically confirmed cases revealed that 66.18% (n = 544) of them fell under the category of primary ICT, 30.90% (n = 254) were metastatic ICTs in nature, and the remaining 2.92% (n = 24) were NVOL. Out of the 543 primary ICT cases, 57.64% (n = 313) were malignant in nature, while the other 42.36% (n = 230) were benign (Figure 1).

Further analysis of primary ICTs revealed that from the 544 cases, 47.24% (n = 257) were tumors from the broad family of glial tumors, including embryonal tumors with multilayered rosettes (ETMLR) (formerly known as primitive neuroectodermal tumor - PNET) and medulloblastoma types of tumors, while the other 52.76% (n = 286) were attributed to meningioma – 39.15% (n = 213), pituitary adenoma – 8.27% (n = 45), neurinoma – 2.21% (n = 12), primary CNS lymphoma – 1.29% (n = 7), hemangiopericytoma – 0.74% (n = 4), cranial chordoma – 0.55% (n = 3), pineocytoma – 0.18% (n = 1), myofibroblast tumor 0.18% (n = 1), and primary intracranial leiomyosarcoma – 0.18% (n = 1).

From the 257 cases of tumors in the glial group, 87.16% (n = 224) were attributed to tumors of astrocytic origin, while the remaining cases were attributed to ependymoma 3.89% (n = 10), oligodendroglioma 3.11% (n = 8), subependymoma 1.95% (n = 5), medulloblastoma 1.17% (n = 3), ETMLR 1.17% (n = 3), oligoastocytoma 0.78% (n = 2), and choroid plexus papilloma 0.78% (n = 2).

Analysis of the 224 tumors with astrocytic origin showed the following distribution: glioblastoma multiforme 81.70% (n = 183), astrocytoma WHO Grades I-II 10.71% (n = 24), anaplastic astrocytoma WHO Grade III 6.25% (n = 14), and gliosarcoma 1.34% (n = 3).

**Figure 2:**
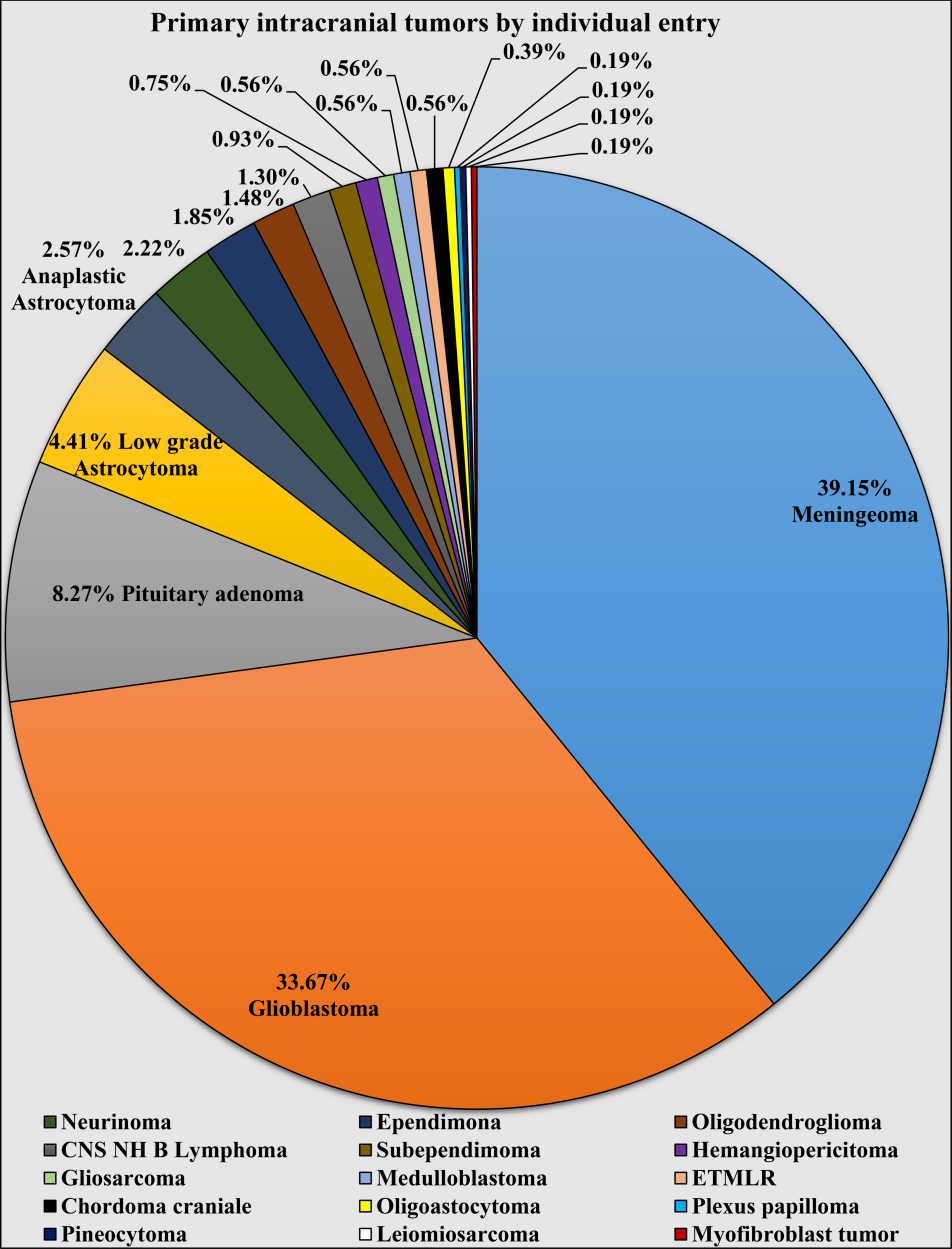
Incidence of individual histologically confirmed types of primary intracranial tumors. CNS NH B lymphomas - central nervous system non-Hodgkin lymphoma; ETMLR - embryonal tumor with multilayered rosettes

Therefore, the most common primary ICT was meningioma (n = 213), accounting for a total of 25.91% of all ICTs and 39.15% of all primary ICTs. The second most common primary ICT and most common malignant primary ICT was glioblastoma multiforme (n = 183), accounting for a total of 22.26% of all ICTs, 33.64% of all primary ICTs, 71.21% of all glial tumors, and 81.70% of all astrocytic tumors. Other common primary entries included astrocytoma WHO Grades I-II, astrocytoma WHO Grade III, and neurinoma. The total incidence of individual types of ICTs are shown in Figure 2; their relations to all primary ICTs, metastatic ICTs, and respected subtypes are shown in Table 1.

**Table 1:** Incidence of Occurrence of Histologically Confirmed Primary and Metastatic Intracranial Tumors and Their Association to Their Individual Groups, Types, and Subtypes WHO: World Health Organization; ETMLR: embryonal tumor with multilayered rosettes; CNS: central nervous system; NVOL: non-tumor volume-occupying lesions

Primary intracranial tumors:	n =	Intracranial tumors %	Primary tumors %	Glial tumors %	Astrocytic tumors %
_Glioblastoma_	_183_	_22.26%_	_33.64%_	_71.21%_	_81.70%_
_Astrocytoma WHO Grades I-II_	_24_	_2.92%_	_4.41%_	_9.34%_	_10.71%_
_Astrocytoma WHO Grade III_	_14_	_1.70%_	_2.57%_	_5.45%_	_6.25%_
_Gliosarcoma_	_3_	_0.36%_	_0.55%_	_1.17%_	_1.34%_
_Ependymoma_	_10_	_1.22%_	_1.84%_	_3.89%_	
_Oligodendroglioma_	_8_	_0.97%_	_1.47%_	_3.11%_	
_Subependymoma_	_5_	_0.61%_	_0.92%_	_1.95%_	
_Medulloblastoma_	_3_	_0.36%_	_0.55%_	_1.17%_	
_ETMLR_	_3_	_0.36%_	_0.55%_	_1.17%_	
_Oligoastrocytoma_	_2_	_0.24%_	_0.37%_	_0.78%_	
_Choroid plexus papilloma_	_2_	_0.24%_	_0.37%_	_0.78%_	
_Meningioma_	_213_	_25.91%_	_39.15%_		
_Pituitary adenoma_	_45_	_5.47%_	_8.27%_		
_Neurinoma_	_12_	_1.46%_	_2.21%_		
_Primary CNS lymphoma_	_7_	_0.85%_	_1.29%_		
_Hemangiopericytoma_	_4_	_0.49%_	_0.74%_		
_Cranial chordoma_	_3_	_0.36%_	_0.55%_		
_Pineocytoma_	_1_	_0.12%_	_0.18%_		
_Leiomyosarcoma_	_1_	_0.12%_	_0.18%_		
_Myofibroblast tumor_	_1_	_0.12%_	_0.18%_		
Total primary intracranial tumors	544				
NVOL:	n =	Intracranial tumors %	NVOL %		
_CNS abscess_	_11_	_1.34%_	_45.83%_		
_Epidermoid cyst_	_7_	_0.85%_	_29.17%_		
_Colloid cyst_	_3_	_0.36%_	_12.50%_		
_Langerhans histiocytosis_	_1_	_0.12%_	_4.17%_		
_Cholesterol granuloma_	_1_	_0.12%_	_4.17%_		
_Demyelinating pseudotumor_	_1_	_0.12%_	_4.17%_		
Total NVOL	24				
CNS metastatic tumors:	n =	Intracranial tumors %	Metastatic tumors %	Pulmonary metastases %	
_Pulmonary adenocarcinoma_	_70_	_8.52%_	_27.56%_	_58.82%_	
_Pulmonary squamous cell carcinoma_	_31_	_3.77%_	_12.20%_	_26.05%_	
_Pulmonary small cell carcinoma_	_18_	_2.19%_	_7.09%_	_15.13%_	
_Malignant melanoma_	_20_	_2.43%_	_7.87%_		
_Colorectal adenocarcinoma_	_16_	_1.95%_	_6.30%_		
_Breast cancer_	_15_	_1.82%_	_5.91%_		
_Gastroesophageal adenocarcinoma_	_7_	_0.85%_	_2.76%_		
_Renal clear cell carcinoma_	_7_	_0.85%_	_2.76%_		
_Urinary tract transitional cell carcinoma_	_7_	_0.85%_	_2.76%_		
_Uterine cervix squamous cell carcinoma_	_3_	_0.36%_	_1.18%_		
_Endometrial adenocarcinoma_	_3_	_0.36%_	_1.18%_		
_Prostate adenocarcinoma_	_2_	_0.24%_	_0.79%_		
_Ovarian carcinoma_	_1_	_0.12%_	_0.39%_		
_CNS Leukemic infiltration_	_1_	_0.12%_	_0.39%_		
_Pancreatic adenocarcinoma_	_1_	_0.12%_	_0.39%_		
CNS metastases without a specified primary location:					
_Adenocarcinoma_	_36_	_4.38%_	_14.17%_		
_Squamous cell carcinoma_	_11_	_1.34%_	_4.33%_		
_Neuroendocrine tumors_	_4_	_0.49%_	_1.57%_		
_Alveolar soft part sarcoma_	_1_	_0.12%_	_0.39%_		
Total metastatic intracranial tumors	254				
Total intracranial tumors	822				

The 24 cases of NVOL were distributed as follows: CNS abscessed 45.83% (n = 10), epidermoid cysts 29.17% (n = 7), colloid cysts 12.50% (n = 3), Langerhans histiocytosis 4.17% (n = 1), cholesterol granuloma 4.17% (n = 1), and demyelinating pseudotumor 4.17% (n = 1). The total incidence of individual types of NVOLs and their relatedness to all ICTs are shown in Table 1.

From the 254 tumors of extracranial origin, 79.53% (n = 202) were with a known primary origin, and the primary locus of origin for the remaining 20.47% (n = 52) remained unknown. The primary place of origin was determined with the help of patient histories, image diagnostics readings, and the presence of primary biopsies histologically correlating to the CNS metastases.

From the 202 cases of metastatic ICTs with a known origin, 58.91 % (n = 119) were from the broad category of metastatic lung cancer, 9.90% (n = 20) from metastatic melanoma, 7.92% (n = 16) from metastatic adenocarcinoma originating from the colorectal region, 7.43% (n = 15) from metastatic breast cancer, 3.46% (n = 7) from metastatic adenocarcinoma originating from the gastroesophageal region, 3.46% (n = 7) from metastatic clear cell carcinoma of the kidneys, 3.46% (n = 7) from transitional cell carcinoma of the urinary tract, 1.48% (n = 3) from metastatic squamous cell carcinoma of the uterine cervix, 1.48% (n = 3) from metastatic endometrial adenocarcinoma, 1% (n = 2) from metastatic prostate adenocarcinoma, 0.50% (n = 1) from metastatic ovarian carcinoma, 0.50% (n = 1) from CNS leukemic infiltration, and 0.50% (n = 1) from pancreatic adenocarcinoma. All registered cancer types were present in both males and females without a clear statistical predominance, excluding breast, uterine cervix, ovarian, endometrial, and prostate carcinomas, which were gender-specific but are not of statistical interest due to the gender specificity and organs of origin.

From the 119 metastatic cases that fell under the category of metastatic lung cancer, 84.87% (n = 101) were from the non-small cell cancer category and the other 15.13% (n = 18) were small cell lung cancer metastases. The 101 cases of non-small cell lung cancer metastases were represented as a total of 69.31% (n = 70) of pulmonary adenocarcinoma and 30.69% (n = 31) of pulmonary squamous cell carcinoma.

Of the 52 cases of intracranial metastases with unknown primary location, 69.24% (n = 36) were diagnosed as metastatic adenocarcinoma, 21.15% (n = 11) as metastatic squamous cell carcinoma, 7.69% (n = 4) as metastatic neuroendocrine tumors, and 1.92% (n = 1) as metastatic alveolar soft part sarcoma. The total incidence of individual types of metastatic ICTs, with regards to their primary location, and relation to all intracranial, metastatic, and pulmonary metastatic tumors is shown in Table 1.

**Figure 3:**
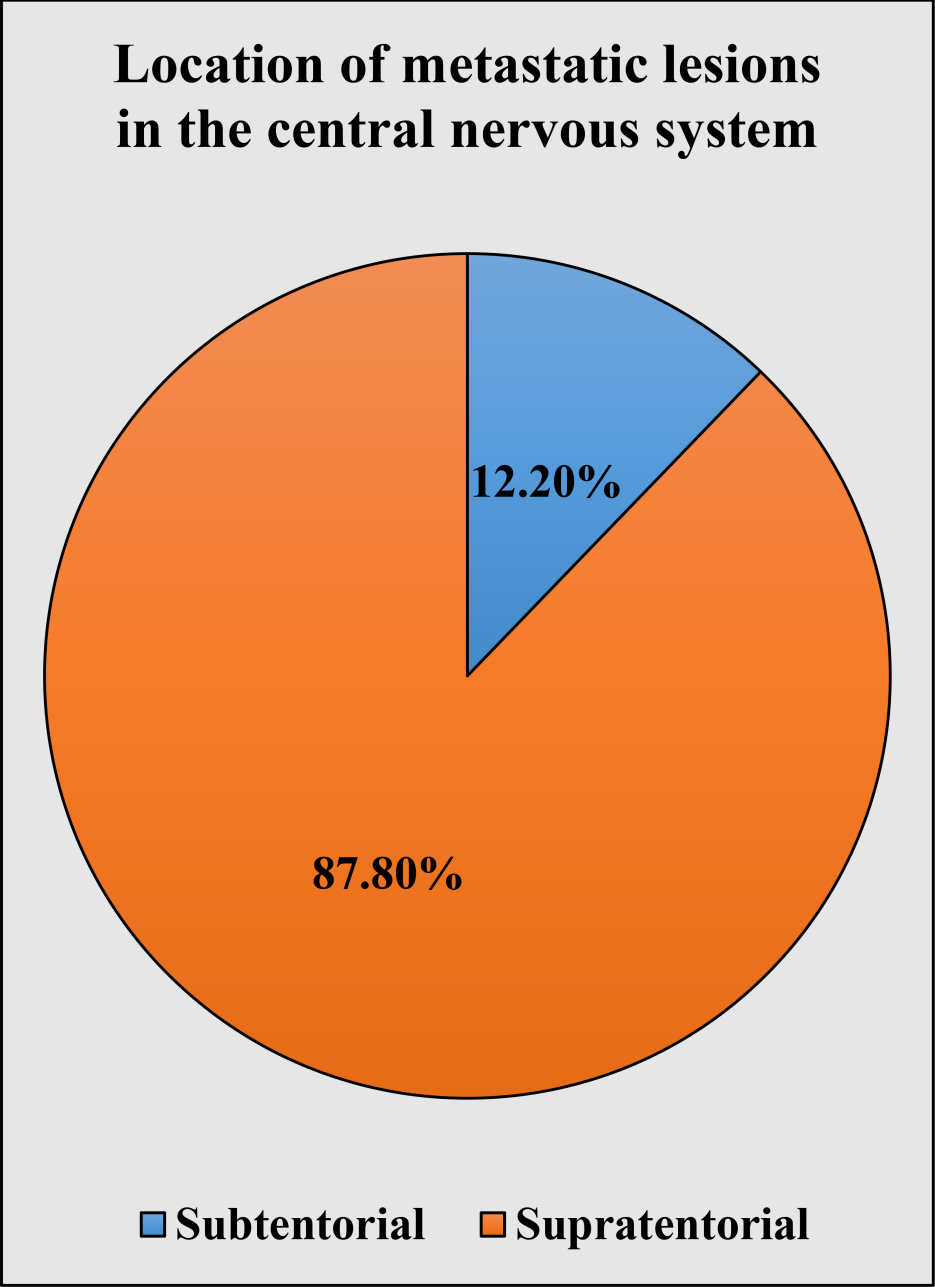
Intracranial location of individual histologically confirmed types of metastatic central nervous system tumors.

A review of the metastatic location of the metastatic tumors (allowed by the biopsy bills filled by the operating neurosurgeons and additional medical documentation) revealed that from all 254 metastatic CNS tumors, 87.80% (n = 223) were supratentorial and the remaining 12.20% (n = 31) were subtentorial (Figure 3). There was no specific tumor type with statistically predominant subtentorial metastatic location.

Therefore, based on the reported findings from the primary ICTs, gliomas represent 31.27% (n = 257 out of 822) of the total cases of ICTs, meningiomas - 25.91% (n = 213 out of 822), pituitary adenomas - 5.47% (n = 45 out of 822), neurinomas - 1.46% (n = 14 out of 822), primary CNS lymphomas represented - 0.85% (n = 7 out of 822), haemangiopericytomas - 0.49% (n = 4 out of 822), cranial chordomas - 0.36% (n = 3 out of 822), pineocytoma - 0.12% (n = 1 out of 822), myofibroblast tumor - 0.12% (n = 1 out of 822), and primary CNS leiomyosarcoma - 0.12% (n = 1 out of 822) (Table 1).

From the NVOL, CNS abscesses represented 1.34% of the cases (n = 11 out of 822), epidermoid cysts - 0.85% (n = 7 out of 822), colloid cysts - 0.36% (n = 3 out of 822), Langerhans histiocytosis - 0.12% (n = 1 out of 822), cholesterol granuloma - 0.12% (n = 1 out of 822), and demyelinating pseudotumor - 0.12% (n = 1 out of 822) (Table 1).

**Figure 4:**
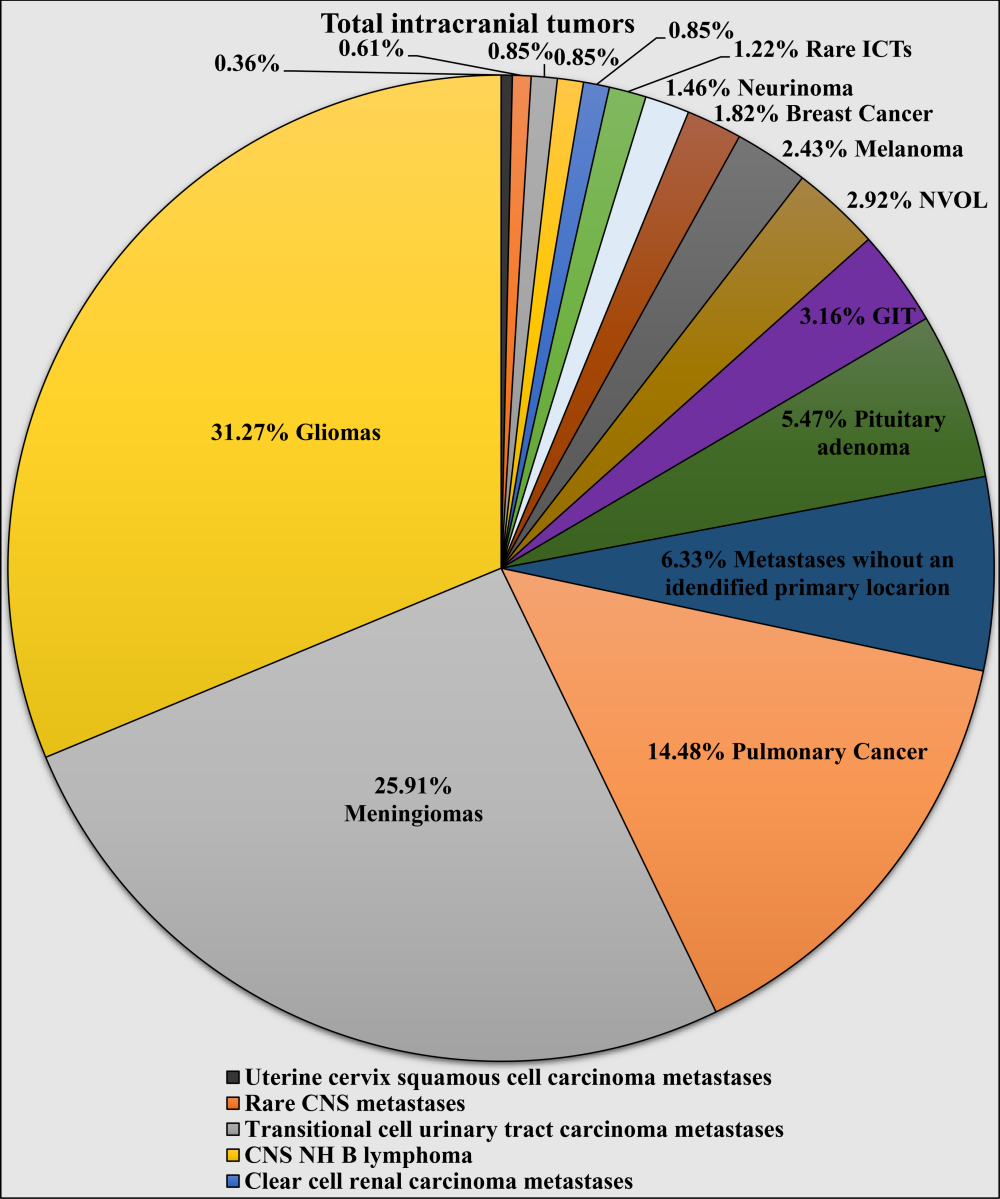
Individual histologically confirmed types of intracranial tumors according to their histological type and location of origin for metastatic lesions. ICTs: intracranial tumors; NVOL: non-volume ocupying lesions; GIT: gastrointestinal tract; CNS: central nervous system; CNS NH B Lymphomas: central nervous system non-Hodgkin lymphoma Note: Rare CNS metastatic entries include ovarian carcinoma, endometrial adenocarcinoma, prostate adenocarcinomas, pancreatic adenocarcinoma, CNS leukemic infiltrations. NVOL includes cerebral abscess, epidermoid and colloid cysts, Langerhans histiocytosis, cholesterol granuloma, and demyelinating pseudotumor. Rare ICTs included cranial chordoma, hemangiopericytoma, pineocytoma, primary intracranial leiomyosarcoma, and myofibroblastic tumor. CNS metastases without a specified primary location included metastases of common types of cancer, such as squamous cell, adenocarcinoma, and neuroendocrine carcinoma, whose primary location remained unspecified and metastases of the rare oncologic entree, alveolar soft part sarcoma.

From the metastatic lesions, subclassification analysis revealed that pulmonary cancer metastases represented 14.48% (n = 119 out of 822) of the total cases of ICTs, metastatic lesions originating from the gastrointestinal tract - 2.92% (n = 24 out of 822), melanoma metastases - 2.43% (n = 20 out of 822), breast cancer metastases - 1.82% (n = 15 out of 822), clear cell renal carcinoma metastases - 0.85% (n = 7 out of 822), transitional cell urinary tract metastases - 0.85% (n = 7 out of 822), uterine cervix squamous cell carcinoma metastases - 0.36% (n = 3 out of 822), endometrial adenocarcinoma - 0.36% (n = 3 out of 822), prostate adenocarcinoma metastases - 0.24% (n = 2 out of 822), ovarian carcinoma metastases - 0.12% (n = 1 out of 822), leukemic infiltration of the CNS - 0.12% (n = 1 out of 822), and the remaining 6.33% (n = 52 out of 822) of the cases were represented by metastatic lesion only identified as a main histologic malignant type, but their primary location remained statistically unknown (Figure 4, Table 1).

**Figure 5:**
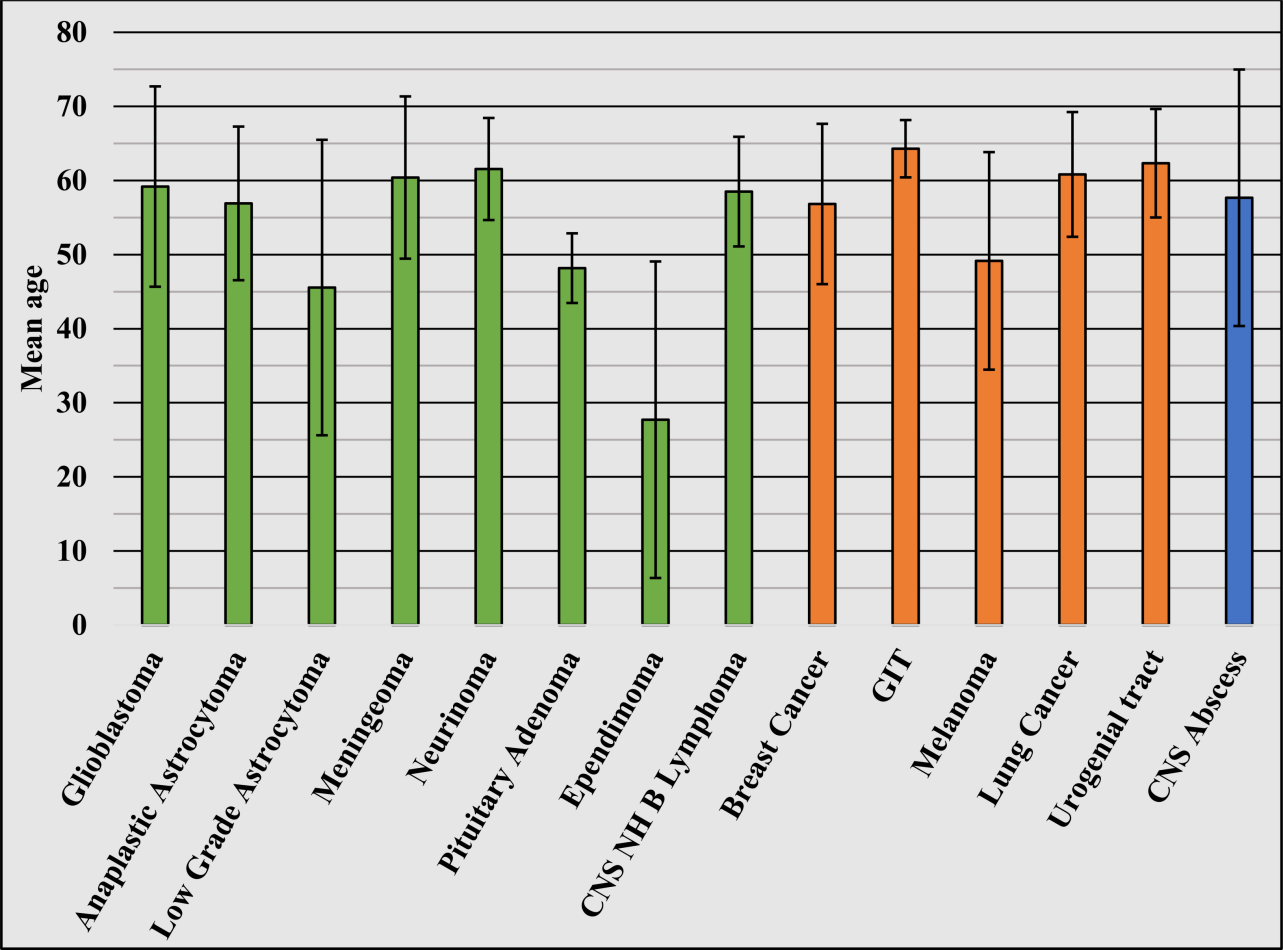
Mean age of diagnosis of the most commonly diagnosed ICTs ICTs: intracranial tumors; CNS: central nervous system; GIT: gastrointestinal tract; CNS NH B lymphomas: central nervous system non-Hodgkin lymphoma

The mean age and male to female ratio for the most common entries are as follows: glioblastoma multiforme (n = 183) mean age: 59.18 years (standard deviation ± 13.52), male to female ratio 1.3:1; astrocytoma WHO Grade III (n = 14) mean age: 56.91 years (standard deviation ± 10.37), male to female ration 1.8:1; astrocytoma WHO Grades I-II (n = 24) mean age: 45.55 years (standard deviation ± 19.94), male to female ratio 1.5:1; meningioma (n = 213) mean age: 60.40 years (standard deviation ± 10.95), male to female ratio 1:1.8; neurinoma (n = 12) mean age: 61.55 years (standard deviation ± 6.89), male to female ratio 1:2.2; pituitary adenoma (n = 45) mean age: 48.17 years (standard deviation ± 4.70), male to female ratio 1.05:1; ependymoma (n = 10) mean age: 27.71 years (standard deviation ± 21.36), male to female ratio 1.1:1; CNS lymphoma (n = 7) mean age: 58.50 years (standard deviation ± 7.40), male to female ratio 1.4:1; breast cancer metastases (n = 15) mean age: 56.83 years (standard deviation ± 10.82), male to female ratio 0:1; gastrointestinal tract (GIT) metastases (n = 32) mean age: 64.29 years (standard deviation ± 3.87), male to female ratio 1.8:1; melanoma metastases (n = 20) mean age: 49.15 years (standard deviation ± 14.98), male to female ratio 1:1; pulmonary metastases (n = 119) mean age: 60.82 years (standard deviation ± 8.42), male to female ratio 3.1:1; urogenital metastases, including gender-specific cancer types (n = 25), mean age: 62.33 (standard deviation ± 7.32), male to female ratio 1.2:1; and CNS abscess (n = 11) mean age: 57.67 years (standard deviation ± 17.31), male to female ratio 2.66:1 (Figure 5).

**Figure 6:**
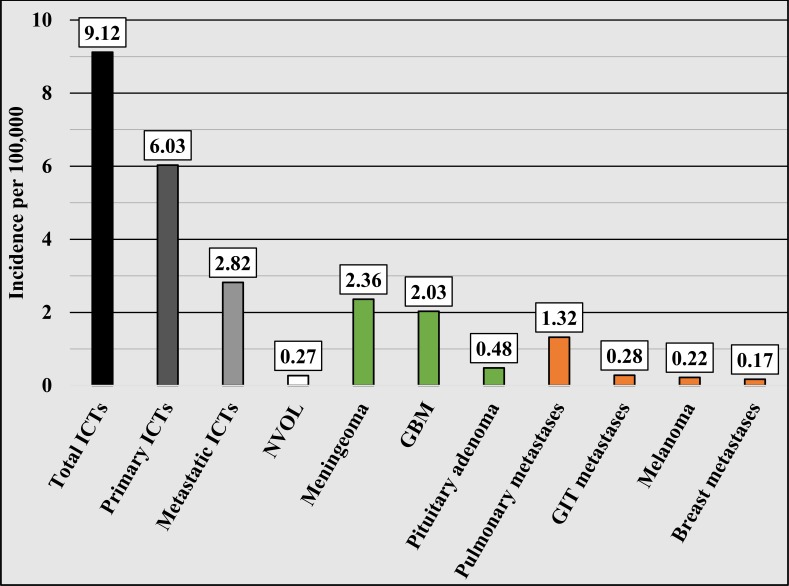
Annual incidence per 100,000 for the most commonly diagnosed ICTs ICTs: intracranial tumors; NVOL: non-tumor volume-occupying lesions: GBM: glioblastoma multiforme; GIT: gastrointestinal tract

Based on the descriptive analysis and the total population of the regions referred to our institutions (1,802,793 people), the annual incidence per 100,000 capita of all ICTs is 9.12, comprised of 6.03 per 100,000 for primary ICTs, 2.82 per 100,000 for metastatic ICTs, and 0.27 per 100,000 for NVOL. The annual incidence of the most commonly diagnosed primary ICTs per 100,000 is 2.36 for meningioma, 2.03 for glioblastoma, and 0.48 for pituitary adenoma. The annual incidence of the most commonly diagnosed metastatic ICTs per 100,000 is 1.32 for lung cancer metastases, 0.28 for GIT metastases, 0.22 for melanoma, and 0.17 for breast cancer metastases (Figure 6).

## Discussion

Most often, neurological deficits are the first sign of an ICT and can be severe and remain permanent, despite the therapeutic actions taken by medical personnel. Segregation between primary and metastatic ICTs is a pivotal moment in the diagnostic process and plays a major role in determining the therapeutic modalities for further treatment and, therefore, plays a key role in managing medical resources. It is the first step towards the application of personalized medicine in the case of ICTs. The knowledge of the incidence and demographics of individual ICT entries are key steps in the preparation, both for the patient intervention and the institutional planning of resources. As such, our report gives a rare glimpse comparing the different types of ICTs and the individual patient demographics for the most common entries.

Similar studies to ours are rarely undertaken [27]. Our results, to our knowledge, are the only ones giving modern insight in such depth to the problem and the first ever to touch on it in our population.

Although favoring the classification of primary ICTs and NVOLs when compared to metastatic ICTs, due to the histological verification criteria, our study reports similar findings to other studies [2, 20, 24, 26-28]. Our findings concur with the total incidence for primary ICTs when compared to similar modern analyses on the topic, giving nearly identical rates for the most commonly diagnosed lesions [2, 27].

To our knowledge, apart from one very recent article, the last published articles that compared primary and metastatic ICTs were published more than three decades ago [26-28]. Our study outnumbers older publications in the total number of cases reviewed, although not in the period of data sampling. However, based on the time gap, these differences could be attributed to advances in neurosurgery and its increased availability for the global population. On the other hand, our results are very close in figures to those reported by Werneck de Carvalho, et al. in Brazil in 2017, despite some variances in study design and populational differences [27]. Nonetheless, the results of all studies concur with ours, stating that primary ICTs are greater in incidence when compared to metastatic ICTs, despite the values ranging from 1.6% to 49% for metastatic ICTs, when compared to our results stating 30.90%.

Since then, due to most metastatic ICTs being diagnosed only radiologically and deemed inoperable, statistical analyses have focused only on comparing either primary or metastatic lesions of the CNS. This is due to methodological incompatibility between the diagnostic criteria for the two groups, making such studies extremely hard to undertake as the criteria, the volume of data analysis, and representation of data would be difficult to compose. Our histological criteria are a drawback in this aspect but nonetheless present a modernized view of the problem, as well as establishing the demographics of patients, previously unreported in our country in such depth. Our results are also consistent with the data from articles investigating the incidence of metastatic ICTs, concurring that the greatest portion of these are attributed to breast, melanoma, lung, and gastrointestinal cancer metastases [1, 23-24].

However, despite the reported data, all authors agree that the total occurrences, and therefore, the statistical percentage of metastatic ICTs, are higher than that of the cases that underwent neurosurgical interventions and were histologically confirmed. This statement is further illustrated mainly by the absence of oncological entries that are known to spread to the CNS but are not present in our study. On the other hand, the presence of some extremely rare entries, such as pineocytoma and primary intracranial leiomyosarcoma, are of great value to the statistical significance of the reported data, as they are rarely present in any statistical report.

To our knowledge, at the present time, no other study has compared the incidence of NVOLs to primary or metastatic ICTs.

## Conclusions

The demographic characteristics of this study establish some aspects of the age and gender ratios for the most commonly diagnosed types of ICTs in our population in a descriptive manner based on histological verification criteria. These findings are the first ever reported in our population and the most detailed report in more than 30 years. Based on our results, primary ICTs represent 66.06% of all histologically verified intracranial lesions, metastatic ICTs represent 30.90%, and only a small fraction of cases are attributed to NVOLs (3.04%). The annual incidence per 100,000 capita of all ICTs was 9.12, comprised of 6.03 for primary ICTs, 2.82 for metastatic ICTs, and 0.27 for NVOL. The most common ICTs diagnosed include glioblastoma, meningioma, and pituitary adenoma for primary ICTs, lung, melanoma, GIT, and breast cancer for metastatic ICTs, and cerebral abscess for NVOL. CNS metastases are mostly supratentorial, accounting for 87.80%, with no statistical evidence of a tumor type predominantly metastasizing in the subtentorial region.
